# Ideal Versus Actual Body Weight for Dosage of Sugammadex in Patients With Body Mass Index Less Than 18.5: A Randomized Controlled Trial

**DOI:** 10.7759/cureus.102536

**Published:** 2026-01-29

**Authors:** Hanae Sato, Tsukasa Uesaka, Takahiro Suzuki, Hajime Iwasaki

**Affiliations:** 1 Department of Anesthesiology, Nihon University School of Medicine, Tokyo, JPN; 2 Department of Anesthesiology and Critical Care Medicine, Asahikawa Medical University, Asahikawa, JPN; 3 Department of Anesthesiology, Medical College of Wisconsin, Milwaukee, USA

**Keywords:** deep neuromuscular block, electromyography, neuromuscular monitoring, sugammadex, underweight patients

## Abstract

Introduction: To date, no study has evaluated the appropriate sugammadex dose in underweight patients. Therefore, we aimed to compare time to recovery from deep neuromuscular block after administration of sugammadex based on either actual or ideal body weight in patients with a body mass index (BMI) of less than 18.5 kg/m^2^.

Methods: Patients with a BMI < 18.5 kg/m^2^ who underwent elective surgery under general anesthesia were enrolled. Anesthesia was induced and maintained with total intravenous anesthesia. Train-of-four (TOF) responses were monitored at the abductor digiti minimi muscle using electromyography following administration of rocuronium 0.6-0.9 mg/kg. An additional dose of rocuronium 0.2 mg/kg was administered to maintain deep neuromuscular block. When the post-tetanic count reached 1 or 2, sugammadex 4 mg/kg was administered, calculated based on either actual or ideal body weight. The primary outcome was the time from sugammadex administration to a TOF ratio ≥ 90%. Data were analyzed using the unpaired* t*-test, with statistical significance defined as p < 0.05.

Results: A total of 32 patients were included in the analysis. The difference between the actual and ideal body weight groups was not statistically significant (185.6 ± 89.5 s vs 147.3 ± 71.0 s; p = 0.19).

Conclusion: There was no significant difference in sugammadex recovery time from deep neuromuscular block between dosing based on actual body weight and dosing based on ideal body weight in underweight patients.

## Introduction

Sugammadex, a modified gamma-cyclodextrin, reverses neuromuscular block by encapsulating steroidal neuromuscular blocking agents, including rocuronium and vecuronium, in a 1:1 complex. The recommended dose of sugammadex depends on the depth of neuromuscular block as determined by quantitative neuromuscular monitoring: 2 mg/kg is recommended when there are at least two train-of-four (TOF) responses [1,2], and 4 mg/kg is recommended when the neuromuscular block is between one post-tetanic count (PTC) and TOF count < 2 [3]. The dose of sugammadex should be calculated based on actual body weight, including for morbidly obese patients with a body mass index (BMI) of 40 kg/m^2^ or higher [4]. This is because administration of sugammadex calculated according to ideal body weight in morbidly obese patients often results in underdosing, leading to inadequate neuromuscular recovery and a greater need for re-administration due to re-onset of neuromuscular block [5].

In contrast, in patients with low body weight, administering sugammadex based on actual body weight may result in underdosing because the calculated dose is lower than that based on ideal body weight. For example, in a patient 170 cm tall and weighing 50 kg (BMI 17.3 kg/m^2^), the dose of sugammadex required to reverse deep neuromuscular block (PTC ≥ 1, TOF count=0) is calculated as 200 mg based on actual body weight, but would be 254 mg if calculated based on ideal body weight. Low body weight is a critical concern in several Asian countries, including India, China, Indonesia, and Japan [6]. To date, the appropriate sugammadex dose in underweight (BMI < 18.5 kg/m^2^) patients is still undefined. The purpose of this study was to compare recovery times from deep neuromuscular block when sugammadex was administered based on either actual body weight or ideal body weight in patients with a BMI of less than 18.5 kg/m^2^. We hypothesized that administration of sugammadex based on ideal body weight would result in faster reversal of deep neuromuscular block than dosing based on actual body weight in underweight patients.

This article was previously presented as a poster at the annual meeting of the Japanese Society of Anesthesiologists, Hokkaido-Tohoku region, on September 13, 2025.

## Materials and methods

Study design

This study was a single-blind, randomized controlled trial conducted at two university hospitals. The study protocol was first approved by the Nihon University Itabashi Hospital Clinical Research Ethics Committee, Tokyo, Japan, on October 4, 2023 (RK-230808-7). It was subsequently approved by the Asahikawa Medical University Research Ethics Committee. The trial was listed in the University Hospital Medical Information Network under registration number jRCT1031230452 on 19 November 2023 (principal investigator: Hanae Sato). All patients provided written informed consent before enrollment. The Strengthening the Consolidated Standards of Reporting Trials (CONSORT) guidelines [7] were followed.

Study population

Patients aged 20 years or older with a BMI < 18.5 kg/m^2^ who underwent surgery under general anesthesia were enrolled in this study. Exclusion criteria included an American Society of Anesthesiologists physical status classification [8] of IV or higher, a serum albumin level less than 3.5 mg/dL, prior allergic reactions to neuromuscular blocking agents, and hepatic (abnormal AST/ALT levels), renal (abnormal serum creatinine levels), or neuromuscular disease listed in the Good Clinical Research Practice guideline [2]. Patients receiving medications that could influence neuromuscular function (e.g., aminoglycoside antibiotics, anticonvulsants, and lithium) were also excluded.

Randomization and blinding

Participants were randomly assigned to either the group receiving sugammadex dosed according to actual body weight or to the group receiving sugammadex dosed according to ideal body weight, using the sealed envelope method. Participants were blinded to group allocation, but investigators were aware of the assignments.

Perioperative management

Upon arrival in the operating room, standard monitors, including electrocardiography, noninvasive blood pressure, and pulse oximetry were applied to all patients. Intravenous access was obtained in either the forearm or on the dorsal venous network of the hand. Following preoxygenation, general anesthesia was induced using propofol (3-4 μg/ml, target-controlled infusion), remifentanil (0.2-0.3 μg/kg/min), and fentanyl (1-2 μg/kg). Anesthesia was maintained with propofol and remifentanil, targeting a bispectral index between 40 and 60. Additional fentanyl was administered as needed. Peripheral temperature was maintained above 35°C using a forced-air warming device for the upper body. End-tidal carbon dioxide was kept within the range of 35-40 mmHg.

Neuromuscular management

An electromyographic neuromuscular monitor (AF-201P, Nihon-Kohden, Tokyo, Japan) was placed to monitor the abductor digiti minimi muscle. After induction of anesthesia, the neuromuscular monitor was calibrated, and rocuronium was administered at a dose of 0.6-0.9 mg/kg. TOF stimulation was applied every 15 seconds. If no TOF response (TOF count = 0) was observed, PTC was measured every six minutes. When the PTC reached 8 or higher, an additional dose of rocuronium (0.2 mg/kg) was administered to achieve PTC = 0. When the PTC was 1 or 2 following PTC = 0, sugammadex was administered at a dose of 4 mg/kg, calculated randomly based on either actual body weight or ideal body weight. To reduce dosing inaccuracies, sugammadex was diluted with normal saline to a concentration of 10 mg/ml. The ideal body weight was calculated as height (m)^ 2^ × 22 [9].

Outcomes of the study

The primary outcome was the time from sugammadex administration to the first TOF ratio (TOFR) ≥ 90% sustained for three consecutive measurements. The secondary outcomes were the supramaximal current, baseline compound muscle action potential, baseline TOFR (measured after calibration and prior to rocuronium administration), final TOFR, serum total protein, and serum albumin.

Sample size and statistical analysis

To determine the sample size, we used previously published data indicating that the mean recovery time from neuromuscular block (defined as TOFR ≥ 90%) after administration of sugammadex 4 mg/kg was 1.9 min [10]. Assuming a 30% reduction in recovery time when sugammadex is administered to underweight patients based on ideal body weight (resulting in a mean recovery time of 1.33 min), the calculated effect size (Cohen’s d) was 1.14, assumed pooled standard deviation of 0.5. A significance level (α) of 0.05 and a power of 0.8 were used for the sample size calculation, resulting in a requirement of 14 patients per group (28 patients in total). To allow for a 15% dropout rate, the final sample size was set at 32 patients.

We used the D’Agostino-Pearson omnibus test to assess whether the data were normally distributed. The unpaired t-test was used for parametric data, and the Mann-Whitney U test was used for nonparametric data. All statistical analyses were performed using GraphPad Prism® version 7.03 (GraphPad Software, Inc., La Jolla, CA), and a P-value of < 0.05 was considered statistically significant.

## Results

A total of 32 patients were enrolled in this study between April 2024 and May 2025. Data from all patients were included in the analysis. Table 1 lists the patient demographics. Mean age was 40.2 ± 19.2 years in the actual body weight group and 47.9 ± 14.7 years in the ideal body weight group (P = 0.21). A total of three men and 29 women were included in the study.

**Table 1:** Patient demographics Data are presented as the mean ± standard deviation or number (%). Differences in outcomes between the two groups were analyzed using the unpaired t-test. SMD, Standardized mean difference; ASA-PS, American Society of Anesthesiologists physical status; BMI, body mass index; NA, not applicable

Characteristic	Actual body weight (n = 16)	Ideal body weight (n = 16)	SMD
Age (y)	40.2 ± 19.2	47.9 ± 14.7	0.45
Height (cm)	158.6 ± 4.9	158.8 ± 9.3	0.03
Weight (kg)	44.4 ± 3.2	43.9 ± 4.9	-0.12
BMI (kg/m^2^)	17.6 ± 0.6	17.4 ± 0.8	-0.33
Men	1 (6%)	2 (13%)	NA
Women	15 (94%)	14 (87%)	NA
ASA-PS classification			
Ⅰ	10 (63%)	11 (69%)	NA
Ⅱ	6 (37%)	5 (31%)	NA
Serum total protein (g/dL)	7.3 ± 0.6	7.2 ± 0.4	-0.03
Serum albumin (g/dL)	4.4 ± 0.3	4.4 ± 0.4	-0.06
Dose of sugammadex (mg)	177.5 ± 12.8	221.4 ± 25.3	NA
Deviation from the sugammadex dose calculated based on actual body weight (mg)	0	45.7 ± 12.7	NA
Type of surgery			
Oral surgery	11 (69%)	5 (31%)	NA
Gastroenterological surgery	3 (19%)	4 (25%)	NA
Breast surgery	1 (6%)	2 (13%)	NA
Orthopedic surgery	0	3 (19%)	NA
Otolaryngology surgery	0	1 (6%)	NA
Gynecological surgery	0	1 (6%)	NA
Neurosurgery	1 (6%)	0	NA

Table 2 presents the results of the primary and secondary outcomes. The primary outcome showed no statistically significant difference in the time to achieve a TOFR of 90% between the actual and ideal body weight groups (185.6 ± 89.5 vs 147.3 ± 71.0 s, P = 0.19). There were no significant differences between groups in terms of any secondary outcome (supramaximal current, baseline compound muscle action potential, baseline TOFR, final TOFR, serum total protein, or serum albumin). Postoperatively, no patients showed signs of residual neuromuscular block or required reintubation.

**Table 2:** Results for primary and secondary outcomes Data are presented as the mean ± standard deviation or median [range]. The unpaired t-test was used for parametric data, and the Mann-Whitney U test was used for nonparametric data. cMAP, compound muscle action potential; TOF, train-of-four

Outcome	Actual body weight (n = 16)	Ideal body weight (n = 16)	P-value
Time to TOF ratio ≥ 0.9 (s)	185.6 ± 89.5	147.3 ± 71.0	0.19
Supramaximal current (mA)	22.1 ± 5.9	23.6 ± 4.4	0.42
Baseline cMAP (mV)	11.0 ± 3.3	12.4 ± 3.0	0.2
Baseline TOF ratio (%)	101.5 [98–103]	101 [100–103]	0.44
Final TOF ratio (%)	100 [98–114]	100 [99–102]	0.97

## Discussion

This study found no statistically significant difference in the time required to achieve a TOFR ≥ 90% between the actual body weight group and the ideal body weight group in underweight patients. We hypothesized that, in underweight patients, dosing based on ideal body weight would result in a shorter recovery to a TOFR ≥ 90% than dosing based on actual body weight; however, this hypothesis was not supported by our findings. Although no statistically significant difference was observed between the groups, the mean time to achieve a TOFR ≥ 90% differed by approximately 38 seconds. This difference may be explained by the mathematical premise that dosing based on actual body weight inherently yields smaller doses in underweight individuals, in contrast to patients with normal weight or obesity. Nevertheless, a 38-second difference in recovery time is unlikely to be clinically meaningful.

The literature on neuromuscular block increasingly centers on obese patient populations, a trend highlighted in recent systematic and bibliometric evaluations [11]. Regarding the dosage of sugammadex, dosing of sugammadex in obese patients is recommended based on actual body weight, not ideal body weight, to prevent underdosing due to increased distribution volume and altered pharmacokinetics [5,12]. However, dosing strategies for underweight patients remain underexplored. Although the differences detected in our study were small, further dose-finding research on sugammadex in underweight patients is warranted to refine dosing recommendations.

A recent dose-finding study of sugammadex found that 87% of patients required less than the recommended dose, whereas 13% required more than the recommended dose; notably, 2% of those patients experienced recurrence of neuromuscular block [13]. This suggests that, although the dosing recommended in the package insert affords a reasonable safety margin, there is substantial interindividual variability in the effect of sugammadex. Residual neuromuscular block can impair swallowing and upper airway muscle function, increasing the risk of upper airway obstruction and aspiration pneumonia [14,15]. Importantly, even mild residual block at a TOFR of 70%-90% is associated with greater pharyngeal dysfunction, upper airway muscle weakness, and an approximate 30% reduction in the hypoxic ventilatory response [16]. Therefore, achieving a TOFR ≥ 95% before extubation is critically important to reduce the incidence of serious postoperative pulmonary complications [17-19]. Given that 13% of patients required more than the standard dose of sugammadex [13], and that underweight patients typically receive smaller doses when dosed by actual body weight, using ideal body weight for dosing in underweight patients may help lower the incidence of underdosing in this group.

It is important to note that excessive sugammadex administration poses significant healthcare economic challenges, as it is considerably more expensive than conventional reversal agents such as neostigmine, with cost differences ranging from $77 to $100 per patient in previous studies [20,21]. However, although ideal body weight-based dosing increases the sugammadex dose in underweight patients, the absolute dose remains markedly lower than that used in obese patients. Nevertheless, when dosing is considered at the vial level, even in underweight patients, those with a height exceeding approximately 151 cm can receive 4 mg/kg within a single 200-mg vial when calculated using actual body weight, whereas ideal body weight-based dosing may require an additional vial. Moreover, the introduction of generic sugammadex, now available in multiple countries, will likely reduce the cost burden.

This study has several limitations. First, the sample size of the study was small. To draw more definitive conclusions regarding the optimal dosing regimen for underweight patients, a dose-finding study with a larger sample size is required. Second, elderly patients were not included in the study. Different results might have been obtained if elderly patients had been included, given their decreased cardiac output and prolonged neuromuscular recovery time [22]. Third, our study included only healthy underweight patients, excluding those with eating disorders or malnutrition associated with hypoalbuminemia, as well as those with sarcopenia and prolonged immobility. These patient subgroups may exhibit altered drug distribution, metabolism, or neuromuscular recovery patterns, which could impact optimal sugammadex dosing requirements [23-25].

## Conclusions

In conclusion, among underweight (BMI < 18.5 kg/m^2^) patients, there was no statistically significant difference in recovery time to a TOFR ≥ 90% from deep neuromuscular block when sugammadex was administered based on actual body weight versus ideal body weight. This study was unable to draw a definitive conclusion on whether actual body weight-based dosing is sufficient for safety or whether ideal body weight-based dosing should be recommended in this population.
